# Lipid Droplets as Metabolic–Epigenetic Signaling Hubs: Interplay Between Phase Separation, Cellular Adaptation, and Disease

**DOI:** 10.3390/cells15141272

**Published:** 2026-07-15

**Authors:** Bin Ai, Xiaodan Chong

**Affiliations:** 1 Department of Precision Medicine, Translational Medicine Center, Naval Medical University, Shanghai 200433, China; aibin@smmu.edu.cn; 2 Clinical Oncology Institute, Translational Medicine Center, Naval Medical University, Shanghai 200433, China

**Keywords:** lipid droplets, phase separation, epigenetics, metabolic reprogramming, ferroptosis, cellular adaptation, cancer metabolism

## Abstract

**Highlights:**

**What are the main findings?**

- This work systematically reveals the functional links among lipid droplet (LD) activity, liquid–liquid phase separation (LLPS), and epigenetic regulation, which jointly govern cellular metabolism and stress responses.
- Aberrant LD remodeling leads to disease-specific metabolic and immune defects across metabolic disorders, neurodegenerative diseases, infectious diseases and malignancies.

**What is the implication of the main finding?**

- It builds a unified conceptual model connecting lipid organelle dynamics, biomolecular condensates, and gene transcriptional control for future mechanistic research.
- Targeting LD and LLPS cascades offers novel therapeutic directions for multiple human diseases, laying reference foundations for subsequent translational and clinical investigations.

**Abstract:**

Lipid droplets (LDs) were long thought to be passive organelles merely for neutral lipid storage. Mounting evidence redefines LDs as dynamic metabolic signaling hubs orchestrating cellular stress adaptation, with multifaceted roles in organelle crosstalk, metabolic reprogramming, redox balance and immune signaling. LD function is tightly intertwined with liquid–liquid phase separation (LLPS) and epigenetic remodeling, bridging cellular metabolism to gene expression and cell fate control. LD biogenesis relies on ER lipid structures, phase-separated protein assemblies and lipid regulatory proteins. Via contacts with multiple organelles, LDs regulate lipid catabolism, ferroptosis, inflammation and chromatin accessibility, while their metabolites directly reshape epigenetic modifications and transcription. LLPS-driven biomolecular condensates further coordinate LD-linked metabolic and stress signaling. Dysregulated LD remodeling mediates metabolic flexibility, immune escape and drug resistance in obesity, metabolic dysfunction-associated steatotic liver disease (MASLD), neurodegeneration, viral infection and cancer. This review summarizes progress in LD biogenesis and metabolism, dissects mechanistic crosstalk between LDs, LLPS and epigenetic control, and outlines LD-driven pathogenic reprogramming across human disorders. We also discuss therapeutic approaches targeting LD and LLPS pathways. Despite promising translational prospects, unresolved mechanistic and clinical hurdles persist. Further research on LD biology will reshape our framework linking metabolism, chromatin regulation and stress adaptation.

## 1. Introduction

Lipid droplets (LDs) are ubiquitous, highly conserved organelles responsible for the storage of neutral lipids, primarily triacylglycerols (TGs) and cholesteryl esters (CEs) [1]. Architecturally unique among cellular organelles, LDs possess a highly hydrophobic neutral lipid core enclosed by a phospholipid monolayer [2]. This monolayer surface hosts a specialized and dynamic proteome, including perilipins, the biogenesis regulator seipin, and members of the cell death-inducing DNA fragmentation factor-α-like effector (CIDE) family [3]. Historically dismissed as inert cytoplasmic repositories solely to energy storage and lipid detoxification, LDs have undergone a fundamental conceptual re-evaluation [4]. Propelled by recent methodological breakthroughs in spatial lipidomics, super-resolution live-cell imaging, and organelle interactome mapping, this re-evaluation redefines LDs as highly dynamic, interactive hubs essential for cellular homeostasis [5,6].

Far from operating in isolation, LDs are extensively integrated into the cellular interactome [7]. They engage in extensive, functionally specialized membrane contact sites with the endoplasmic reticulum (ER), mitochondria, lysosomes, and peroxisomes [8,9]. Through these membrane contact sites, LDs not only store lipids but also orchestrate the flux of metabolic intermediates [1,10,11]. This physical coupling empowers cells to regulate fatty acid oxidation, buffer lipotoxicity, neutralize reactive oxygen species (ROS), and modulate inflammatory signaling during periods of nutrient deprivation or microenvironmental insult [12].

Recently, LD regulation has emerged as conceptually linked to two active frontiers in molecular biology: liquid–liquid phase separation (LLPS) and epigenetic remodeling [13]. From a biophysical perspective, the initial nucleation of LDs is increasingly modeled as an LLPS process, wherein neutral lipids phase-separate within the hydrophobic core of the ER membrane to form nascent droplets [14]. Furthermore, many LD-resident proteins—particularly those containing intrinsically disordered regions (IDRs)—exhibit condensate-like behavior [15]. These biomolecular condensates facilitate the spatiotemporal compartmentalization of biochemical reactions, thereby regulating LD maturation, homotypic membrane fusion, and adaptive responses to stress [7,16].

Parallel to these biophysical mechanisms, LDs exert significant biochemical control over nuclear gene expression through an emerging metabolic-epigenetic axis [17]. The controlled mobilization of LD lipid stores provides a regulated supply of metabolites—including acetyl-CoA, S-adenosylmethionine (SAM), and NAD+—which serve as essential cofactors and substrates for chromatin-modifying enzymes [6]. By controlling the availability of these metabolites, lipolytic metabolism at LDs modulates histone acetylation, DNA methylation, and sirtuin-mediated deacetylation [18]. This metabolic-epigenetic coupling acts as a molecular integrator, converting metabolic fluctuations into stable, long-term transcriptional programs that enable adaptation to hypoxia, nutrient deprivation, and oncogenic stress [19,20]. Consequently, the structural and functional integrity of LDs is central to adaptive reprogramming and human health. Aberrant LD remodeling is recognized as a driving hallmark in numerous pathologies, exacerbating metabolic dysfunction-associated steatotic liver disease (MASLD, the newly adopted nomenclature for non-alcoholic fatty liver disease or NAFLD), obesity, diabetes, and neurodegenerative disorders, while also being exploited by viral pathogens to facilitate replication [4,21]. In the context of oncology, hijacked LD metabolism endows aggressive tumor cells with remarkable metabolic plasticity, evasion of host immunity, and profound resistance to ferroptosis [22,23].

This review aims to synthesize recent advances in LD biogenesis and physiological function, with particular emphasis on the tripartite interplay among LD metabolism, biophysical phase separation, and epigenetic reprogramming. Finally, we discuss how these integrated mechanisms contribute to pathogenesis and evaluate the translational potential of therapeutic strategies targeting LD-associated pathways.

## 2. Molecular Basis of Lipid Droplet Biogenesis

### 2.1. ER-Derived Initiation of Lipid Droplets

**Figure 1:**
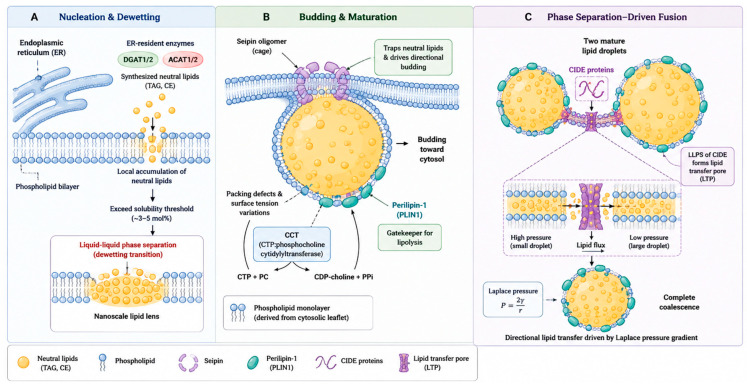
The Spatiotemporal Model of Lipid Droplet (LD) Biogenesis, Maturation, and LLPS-Driven Coalescence. (**A**) Nucleation & Dewetting: LD biogenesis initiates within the endoplasmic reticulum (ER). Neutral lipids (TG/CE) synthesized by ER-resident enzymes (e.g., DGAT) accumulate between the bilayer leaflets. Upon exceeding their solubility threshold, neutral lipids undergo spontaneous liquid–liquid phase separation (LLPS)—a dewetting transition—to form nanoscale lipid lenses. (**B**) Budding & Maturation: Seipin oligomers form a functional cage at the ER-LD contact site, physically trapping neutral lipids and facilitating directional budding of the LD toward the cytosol. Concurrently, localized surface tension variations and packing defects expose the hydrophobic core, which dynamically recruits CTP: phosphocholine cytidylyltransferase (CCT) to synthesize phosphatidylcholine (PC), stabilizing the expanding phospholipid monolayer. The mature monolayer is further decorated with gatekeeper proteins such as Perilipin-1 (PLIN1). (**C**) Phase Separation-Driven Fusion: At LD-LD contact sites, CIDE family proteins undergo LLPS to establish a highly stable lipid transfer pore. Directional lipid flux is thermodynamically driven by the Laplace pressure gradient, facilitating the transfer of neutral lipids from the smaller droplet (high pressure) into the larger one (low pressure) until complete coalescence is achieved. Abbreviations: DGAT, diacylglycerol acyltransferase; TG, triacylglycerol; CCT, CTP:phosphocholine cytidylyltransferase; PLIN1, Perilipin-1; LLPS, liquid liquid phase separation; CIDE, cell death-inducing DFFA-like effector. Arrows throughout indicate sequential steps of formation, physical effects such as phase separation and pressure gradients, and key molecular regulatory interactions.

Lipid droplets (LDs) biogenesis initiates at specialized microdomains within the endoplasmic reticulum (ER). This process is driven by the localized accumulation of newly synthesized neutral lipids—predominantly triacylglycerols (TGs) and cholesteryl esters (CEs)—between the leaflets of the ER membrane [24]. Neutral lipid synthesis is catalyzed by ER-resident acyltransferases, notably diacylglycerol acyltransferases (DGAT1/2) [25] and acyl-CoA:cholesterol acyltransferases (ACAT1/2) [26]. Upon exceeding their solubility threshold within the phospholipid bilayer, these hydrophobic neutral lipids undergo LLPS, forming nanoscale lipid lenses (Figure 1A) [27]. Recent models suggest that the core mechanisms of LD biogenesis—spanning from initial neutral lipid phase separation to directional budding—are broadly conserved across droplets of varying sizes and are dynamically regulated by the ER membrane architecture and local lipid saturation [7].

The spatiotemporal dynamics of these nascent lenses are regulated by specific ER membrane proteins (Table 1). The ER integral membrane protein seipin is essential for this process, assembling into oligomeric ring-shaped structures at nascent LD sites [4]. These seipin complexes act as molecular scaffolds that sequester diffusing neutral lipids, thereby facilitating directional lens expansion and preventing ectopic budding [28]. In parallel, fat storage-inducing transcript (FIT) proteins, such as FIT2, partition diacylglycerol and mediate local lipid remodeling to support nascent LD maturation [29].

Driven by continuous neutral lipid synthesis and local membrane curvature changes, enlarging lipid lenses subsequently bud from the ER membrane (Figure 1B). This budding is highly directional, directed predominantly toward the cytosol rather than into the ER lumen [30,31]. This asymmetry arises from differences in membrane surface tension and spontaneous curvature between the cytosolic and luminal leaflets [32]. Consequently, mature LDs are released as independent organelles enclosed by a phospholipid monolayer primarily derived from the ER cytosolic leaflet, enabling dynamic interactions with cytosolic metabolic enzymes [2,7].

The structural integrity and morphological maturation of newly budded LDs depend critically on local phospholipid homeostasis (Figure 1B) [33]. The dynamic balance of phospholipids, particularly phosphatidylcholine (PC) and phosphatidylethanolamine (PE), determines monolayer surface tension [34]. During rapid LD expansion, the increased surface area generates transient packing defects that expose the hydrophobic neutral lipid core [35]. These structural defects are recognized by CTP: phosphocholine cytidylyltransferase (CCT), the rate-limiting enzyme in PC synthesis, which translocates to the LD surface to upregulate local PC production [36]. Perturbations in this phospholipid balance compromise LD stability, promoting aberrant coalescence and triggering lipotoxic stress [27,36].

### 2.2. Key Regulators of LD Formation

#### 2.2.1. Seipin Complex and Early Nucleation

Seipin is a highly conserved integral endoplasmic reticulum (ER) membrane protein that functions as a key regulator of lipid droplet (LD) nucleation and maturation [37]. Recent structural studies reveal that seipin assembles into an oligomeric ring at ER membrane sites where LDs nucleate [38]. This oligomeric ring functions as a nanoscale lipid diffusion trap, sequestering diacylglycerols (DAG) and triacylglycerols (TG) to facilitate directional lens expansion. By establishing a physical boundary, seipin channels the unidirectional flux of newly synthesized neutral lipids into the growing LD while preventing their retrograde diffusion into the ER membrane [14,38]. Loss-of-function mutations in the BSCL2 gene (encoding seipin) compromise this barrier function, resulting in generalized congenital lipodystrophy characterized by ectopic lipid deposition and aberrant microdroplet clustering [39]. Furthermore, recent structural studies indicate that seipin does not merely act as a static barrier but rather forms oligomeric scaffolds that dynamically recruit regulatory proteins, such as Ldo45, to adapt to fluctuations in intracellular neutral lipid availability [40].

#### 2.2.2. Perilipins and Lipolytic Gating

The perilipin (PLIN) family (PLIN1–PLIN5) constitutes the predominant proteins component of the LD monolayer, regulating lipid storage and mobilization [41]. PLINs contain tandem repeats of amphipathic α-helices that anchor them stably into the phospholipid monolayer of mature LDs [42]. Recent biophysical models indicate that PLINs modulate LD surface tension and regulate the recruitment of lipases, such as adipose triacylglycerol lipase (ATGL) and hormone-sensitive lipase (HSL) [43]. In the basal state, PLIN1 acts as a barrier against unregulated lipolysis. Upon β-adrenergic stimulation, site-specific phosphorylation of PLIN1 triggers extensive conformational remodeling, dissociating the co-activator CGI-58, which subsequently activates ATGL to initiate regulated fatty acid mobilization [44]. Furthermore, tissue-specific isoforms, such as PLIN5 enriched in the oxidative tissues, tether LDs to mitochondria to facilitate lipid transfer and β-oxidation [45].

#### 2.2.3. CIDE Family and Phase-Separated Lipid Transfer

The cell death-inducing DFF45-like effector (CIDE) family—comprising CIDEA, CIDEB, and CIDEC/FSP27—mediates a specialized LD fusion mechanism essential for generating large unilocular LDs characteristic of mature white adipocytes [7]. Recent studies demonstrate that CIDEC and CIDEA are specifically enriched at LD–LD contact interfaces, where they undergo LLPS to form protein condensates (Figure 1C) [46]. This localized phase separation drives the assembly of a lipid transfer pore (LTP) between adjacent LDs [47]. Through this pore, directional lipid transfer is thermodynamically driven by the Laplace pressure gradient, directing the flux of neutral lipids from the smaller droplet (higher internal pressure) into the larger one (lower pressure) until complete coalescence [48,49].

#### 2.2.4. FIT Proteins and Lipid Remodeling

Fat storage-inducing transmembrane (FIT) proteins, particularly FIT2, cooperate with the aforementioned regulators to remodel ER membrane lipid composition during early biogenesis. Acting as an acyl-CoA diphosphatase, FIT2 hydrolyzes local acyl-CoAs to regulate DAG levels, thereby modulating local membrane curvature and preventing lipid-induced ER stress during LD biogenesis [29]. Concurrently, Rab GTPases and coat protein complex I (COPI) machinery regulate ER membrane budding dynamics, ensuring that LD biogenesis is tightly coupled to cellular nutrient availability and metabolic demands [14,29].

### 2.3. Physical Basis of LD Formation and Phase Separation

The *de novo* biogenesis of lipid droplets (LDs) is governed by thermodynamic principles of LLPS occurring within the endoplasmic reticulum (ER) membrane [50]. Neutral lipids, such as triacylglycerols (TGs), exhibit high hydrophobicity and have a low solubility limit (estimated at approximately 3–5 mol%) within the ER phospholipid bilayer [15]. Once local neutral lipid biosynthesis exceeds this critical concentration threshold, the lipid mixture minimizes its free energy through a spontaneous demixing process, often described as a dewetting transition [51]. This segregates neutral lipids from the amphipathic bilayer environment, nucleating nanoscale phase-separated lipid lenses. The subsequent morphological evolution and stabilization of these nascent lenses are determined by a precise balance of interfacial biophysics [52]. Phospholipid composition, degree of acyl chain saturation, and monolayer surface tension regulate the energy required to deform the ER membrane [14]. As the lipid lens expands, changes in membrane line tension and spontaneous curvature drive the directional budding of the LD toward the cytosol, ensuring structural stability while minimizing hydrophobic surface exposure. Beyond the phase separation of constituent lipids, recent biophysical advances have redefined LDs as dynamic platforms for biomolecular condensates [7,13]. Many LD-associated proteins contain intrinsically disordered regions (IDRs) that undergo LLPS at the lipid-water interface [49]. These local protein condensates form functional microdomains that alter local monolayer dynamics. For instance, the fusion and coalescence of mature LDs are not random collisions but rather highly regulated processes governed by Laplace pressure gradients, whereby internal pressure differentials between droplets of varying radii drive lipid flux through phase-separated protein pores [48]. Consequently, LDs represent a unique class of organelles that integrate the physicochemical properties of lipid-driven phase separation and protein condensates [53].

## 3. Lipid Droplets as Metabolic Signaling Platforms

### 3.1. Lipid Buffering and Protection Against Lipotoxicity

**Figure 2:**
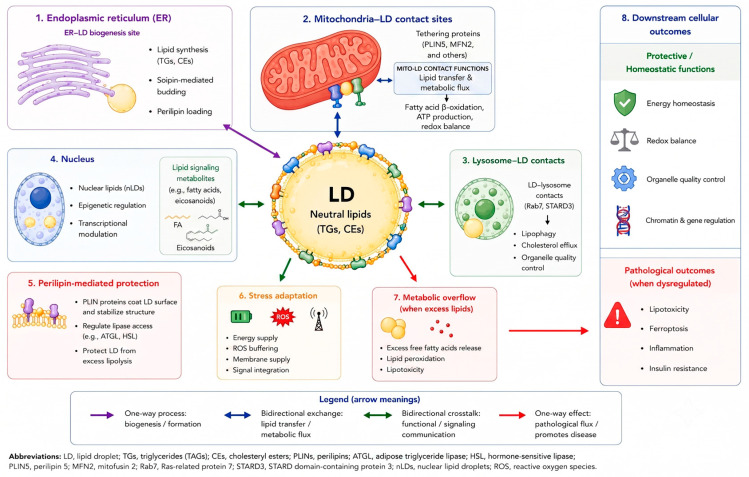
Intracellular Interaction Network of Lipid Droplets and Their Impact on Cellular Homeostasis and Pathology. This figure systematically delineates the role of lipid droplets (LDs) as dynamic central hubs within the cellular network, highlighting their complex functional interplay with various organelles. LD biogenesis originates at the endoplasmic reticulum (ER) via Seipin-mediated budding to store neutral lipids, including triglycerides (TGs) and cholesteryl esters (CEs). The surface of these droplets is coated with perilipin proteins, which stabilize the organelle structure and restrict lipase accessibility to prevent unregulated lipolysis. Functioning beyond mere storage, LDs engage in active bidirectional crosstalk with other cellular compartments. They establish contact sites with mitochondria to facilitate lipid transfer and fatty acid β-oxidation for ATP production, while their interactions with lysosomes govern lipophagy and organelle quality control. Additionally, LDs supply lipid signaling metabolites that modulate epigenetic and transcriptional programs in the nucleus. Under physiological conditions, LDs support stress adaptation by providing energy substrates and buffering reactive oxygen species (ROS). However, excessive lipid accumulation leads to metabolic overflow, triggering a unidirectional pathological cascade. Consequently, downstream cellular outcomes are bifurcated: homeostatic regulation promotes protective functions such as energy and redox balance, whereas dysregulation and subsequent lipid peroxidation drive pathological states, including lipotoxicity, ferroptosis, inflammation, and insulin resistance.

Lipid droplets (LDs) serve as protective compartments against lipotoxic stress by partitioning lipids according to saturation status [54]. In energy-rich states, the incorporation of excess free fatty acids (FFAs) into triacylglycerols within LDs prevents the accumulation of lipotoxic intermediates such as ceramides and diacylglycerols, which can provoke endoplasmic reticulum (ER) stress and apoptotic signaling [55]. Recent studies indicate that this buffering is selective and depends on fatty acid saturation; for instance, metabolic sensors preferentially respond to saturated fatty acids over monounsaturated variants to drive differential lipid storage pathways in tissues such as the myocardium [56]. Furthermore, adaptive cellular responses upregulate LD biogenesis to sequester oxidation-sensitive polyunsaturated fatty acids (PUFAs). The stabilization of stress-responsive transcription factors, such as NRF2, enhances the expression of LD-associated proteins including G0S2, which inhibits adipose triacylglycerol lipase (ATGL) to restrict fatty acid release, thereby preventing lipid peroxidation and lipotoxicity [57]. Collectively, these observations support the role of LDs as dynamic platforms coordinating stress adaptation and metabolic homeostasis (Figure 2).

### 3.2. LD–Mitochondria Coupling and Energy Metabolism

The physical and functional tethering of LDs to mitochondria establishes a critical metabolic interface regulating cellular bioenergetics (Figure 2). Rather than passive proximity, these membrane contact sites are highly regulated microdomains organized by tethering complexes (e.g., PLIN5 and mitofusin 2) that facilitate the direct transfer of fatty acids for β-oxidation [58,59]. During nutrient deprivation or increased energy demand, this coupling ensures continuous fuel delivery to support oxidative phosphorylation while limiting the cytosolic diffusion of lipotoxic intermediates. Recent evidence indicates that the dynamic remodeling of LD-mitochondrial membrane contact sites is a major contributor to metabolic diseases, such as obesity and diabetes, where chronic lipid overload disrupts mitochondrial bioenergetics [1]. Pathological FA overload at these contact sites can overwhelm mitochondrial oxidative capacity, leading to electron leak from the electron transport chain, excessive reactive oxygen species (ROS) generation, and subsequent mitochondrial dysfunction [1]. These LD-mitochondria membrane contact sites (MCSs), regulated by various Rab GTPases, are dynamically generated in response to metabolic or immune cues to ensure efficient delivery of LD lipids for substrate utilization and to prevent cytosolic lipotoxicity [60]. Consequently, LDs function as dual regulators of redox homeostasis, balancing efficient energy extraction against oxidative stress.

In parallel with mitochondrial coupling, LDs also establish critical contact sites with peroxisomes—mediated by specific tethering factors such as ABCD1 and Spastin—to facilitate the oxidation of very-long-chain fatty acids (VLCFAs) and further mitigate oxidative stress.

### 3.3. LD–ER Interactions

The ER serves as the primary site for LD biogenesis, representing the core platform of the LD-ER interactome [7]. The formation of nascent LDs involves the accumulation of newly synthesized neutral lipids within the hydrophobic core of the ER bilayer, forming lipid lenses that bud into the cytosol [4]. This budding process is regulated by ER-resident protein scaffolds, notably seipin, which coordinates neutral lipid lens organization and maintains the architectural integrity of the resulting LDs [4]. Even after maturation, LDs maintain structural continuity with the ER through specialized membrane contact sites (Figure 2). These contact sites are indispensable for local triacylglycerol synthesis at the LD surface, bidirectional phospholipid exchange, and protein trafficking [61]. During cellular stress, the accumulation of unfolded proteins triggers the unfolded protein response (UPR), which enhances ER-LD contact sites to expand the LD pool, thereby restoring ER membrane homeostasis and sequestering lipotoxic intermediates [15].

Furthermore, in specialized secretory cells such as hepatocytes and enterocytes, the ER dictates a critical metabolic bifurcation: the partitioning of newly synthesized neutral lipids between cytosolic lipid droplets (cLDs) and luminal lipoproteins (e.g., VLDL and chylomicrons) [62]. This topological decision is heavily regulated by the microsomal triacylglycerol transfer protein (MTP), which shuttles lipids into the ER lumen to lipidate apolipoprotein B (ApoB). When luminal lipoprotein assembly is saturated, genetically impaired, or pharmacologically inhibited, excess neutral lipids are rapidly shunted toward the cytosolic ER leaflet, driving robust cLD biogenesis and contributing significantly to cellular steatosis. Conversely, cLDs are not entirely isolated from the secretory pathway; they can undergo targeted lipolysis to mobilize fatty acids that are subsequently re-esterified and channeled back into the ER lumen for VLDL assembly [63,64]. This highlights a highly dynamic, bidirectional crosstalk between intracellular lipid storage and systemic lipoprotein transport, dictated by the metabolic state of the ER.

### 3.4. Lipophagy and LD–Lysosome Interactions

Lipophagy, the selective autophagic degradation of LDs, serves as a crucial regulatory mechanism for lipid mobilization and organelle turnover (Figure 2) [65]. Under nutrient-deprived conditions, the delivery of LDs to lysosomes via autophagy-related proteins (ATGs) and Rab GTPase networks facilitates the breakdown of triacylglycerols by lysosomal acid lipases, sustaining critical energy demands [66]. Beyond energy provision, lipophagy functions as an essential quality control mechanism to clear oxidatively damaged LDs. Impairment in lipophagic flux leads to aberrant intracellular lipid accumulation, driving the pathogenesis of metabolic diseases including atherosclerosis and MASLD [65]. Conversely, in aggressive malignancies, tumor cells frequently exploit lipophagy to maintain metabolic plasticity, ensuring a supply of membrane lipids and ATP required for rapid proliferation and metastasis under hypoxic or nutrient-poor microenvironments [67].

### 3.5. Lipid Droplets and Ferroptosis

Ferroptosis, an iron-dependent form of regulated cell death driven by uncontrolled phospholipid peroxidation, is closely linked to intracellular lipid compartmentalization [68]. LDs have recently emerged as critical regulators of cellular sensitivity to ferroptosis (Figure 2). The incorporation of PUFAs into cellular membranes by enzymes such as acyl-CoA synthetase long-chain family member 4 (ACSL4) strongly sensitizes cells to ferroptosis [69]. As a protective mechanism, cells can redirect these oxidizable PUFAs into the neutral lipid core of LDs, sequestering them from membrane lipid peroxidation cascades and conferring ferroptosis resistance [70]. Notably, excessive mobilization of these stored PUFAs can rapidly generate lipid peroxides and resensitize cells to ferroptosis. Within the tumor microenvironment, cancer cells frequently co-opt altered LD dynamics to sequester these oxidation-sensitive lipids, evade ferroptosis, and maintain viability under therapeutic pressure [68,69].

### 3.6. Lipid Droplets in Immune Metabolism

LDs are increasingly recognized as signaling platforms regulating immune cell fate, functional polarization, and inflammatory responses (Figure 2). In innate immune cells, particularly macrophages, LDs serve as dynamic platforms for the synthesis of eicosanoids and other bioactive lipid mediators essential for modulating inflammation [71].The critical role of lipid-associated macrophages (LAMs)—a specialized immune subset characterized by extensive LD accumulation and lipid handling capacity—has been identified in coordinating inflammatory responses during metabolic diseases, positioning LAMs as key regulators of meta-inflammation [72]. Furthermore, within the immunosuppressive tumor microenvironment, LD accumulation in tumor-infiltrating immune cells drives their metabolic reprogramming. Such lipid-rich myeloid cells often exhibit impaired anti-tumor effector functions, contributing to tumor progression and immune evasion [73]. In adaptive immunity, LD dynamics regulate the availability of fatty acids required for mitochondrial metabolism during T-cell activation, establishing LDs as integrators of immunity and systemic metabolism [74].

## 4. Phase Separation in Lipid Droplet Biology

### 4.1. Principles of Liquid–Liquid Phase Separation

Liquid–liquid phase separation (LLPS) has emerged as a fundamental physicochemical mechanism driving cellular compartmentalization [75]. Through spontaneous demixing, macromolecules with multivalent interactions—often driven by intrinsically disordered regions (IDRs), specific RNA-binding domains, and weak electrostatic forces—coalesce into dense, membraneless biomolecular condensates while leaving the surrounding milieu dilute [76]. This dynamic segregation enables cells to spatially and temporally coordinate complex biochemical reactions, enhancing reaction kinetics or sequestering sensitive molecules from degradation. Prototypical examples include nucleoli, P bodies, transcriptional condensates, and stress granules (SGs) [77]. Recent biophysical studies indicate that lipid droplets (LDs), despite being conventionally viewed as simple neutral lipid reservoirs, share fundamental thermodynamic and kinetic properties with biomolecular condensates, expanding the understanding of organelle biogenesis [7].

### 4.2. Are Lipid Droplets Phase-Separated Organelles?

LD biogenesis is fundamentally a phase separation event. As newly synthesized neutral lipids, such as triacylglycerols (TGs) and cholesteryl esters (CEs), accumulate within the hydrophobic core of the ER bilayer, they reach a critical thermodynamic threshold [52]. Once local concentrations exceed 5–10 mol%, they undergo spontaneous demixing to form distinct lipid lenses [15]. Unlike classical protein- or nucleic-acid-driven condensates, LDs represent unique lipid-enriched condensates stabilized by a phospholipid monolayer and a specialized surface proteome [78]. Nevertheless, both membraneless condensates and LDs regulate their assembly, coalescence, and dissolution via shared physicochemical principles [7]. This dual nature integrates features of classical membrane-bound organelles and liquid condensates, characterizing LDs as hybrid phase-separated structures [15].

### 4.3. LLPS in LD Assembly and Remodeling

Proteins driving LD assembly also undergo phase separation to determine organelle architecture. Seipin, an essential ER-resident scaffold protein, assembles into oligomeric rings at ER-LD junctions, facilitating the liquid–liquid phase transition of neutral lipids [15]. By trapping diacylglycerols and TGs, seipin lowers the energy barrier for lipid phase separation and ensures directional budding of the nascent droplet [15]. Beyond biogenesis, the LD surface proteome exhibits liquid-like dynamics. Scaffolding proteins such as perilipins partition into these interfaces, regulating droplet fusion, expansion rates, and lipolysis [79]. During environmental stress—such as hypoxia or nutrient deprivation—these phase-dependent protein interactions rapidly restructure the LD surface, enabling adaptive remodeling of lipid stores in response to acute metabolic stress [52].

### 4.4. LD-Associated Proteins Undergoing Phase Separation

Emerging evidence links LDs to the phase behavior of aggregation-prone proteins, specifically those implicated in neurodegenerative diseases and RNA metabolism [13]. Under metabolic or oxidative stress, RNA-binding proteins containing IDRs, including FUS and TDP-43, localize to the LD monolayer [80]. Furthermore, recent studies indicate that lipid droplets can act as nucleation platforms promoting the pathological aggregation of proteins such as alpha-synuclein, which sequesters lipids and disrupts adjacent mitochondrial homeostasis [13]. By acting as functional scaffolds or sponge-like compartments, LDs sequester potentially toxic condensates to mitigate proteotoxicity; however, when dysregulated, they can accelerate pathological protein aggregation. This reveals a bidirectional relationship between lipid metabolism and proteostasis [7].

### 4.5. Crosstalk Between LDs and Stress Granules

The interplay between LDs and canonical biomolecular condensates such as stress granules (SGs) contributes to a coordinated stress response network [81]. Cellular stressors that trigger SG formation—such as viral infections, heat shock, or oxidative stress—concomitantly induce LD accumulation. SGs transiently sequester mRNAs and translation machinery to halt global protein synthesis and preserve energy, while LDs serve as metabolic buffers that sequester lipid peroxides generated during these stress events [82]. Emerging models propose a bidirectional regulatory loop: SG-associated scaffolding proteins (e.g., G3BP1) may associate with LDs or compete for condensate components, whereas metabolic shifts initiated by LD remodeling provide energy substrates and lipid signaling molecules necessary for SG assembly and clearance [83]. This crosstalk connects RNA regulation with lipid compartmentalization [84].

### 4.6. Phase Separation and Metabolic Adaptation

The integration of LLPS mechanisms with LD biology reveals a rapid, reversible mechanism for cellular metabolic adaptation [76]. Because phase transitions are highly sensitive to subtle intracellular changes—such as shifts in pH, salt concentration, and redox state—LLPS enables rapid sensing of metabolic stress [85]. The subsequent phase-separation-driven remodeling of LDs redirects intracellular lipid fluxes, switching energy utilization patterns to promote cell survival [86]. For instance, the shift from mitochondrial oxidation to glycolysis under hypoxia is accompanied by the reorganization of glycolytic enzymes into condensates alongside LD accumulation. Ultimately, the interconnected sequence of “stress → phase separation → LD remodeling → metabolic rewiring” acts as a pivotal adaptive axis, with profound implications for treating metabolic disorders, infectious diseases, and cancer [87].

## 5. Crosstalk Between Lipid Droplets and Epigenetic Regulation

### 5.1. Metabolic Substrates and Epigenetic Remodeling

**Figure 3:**
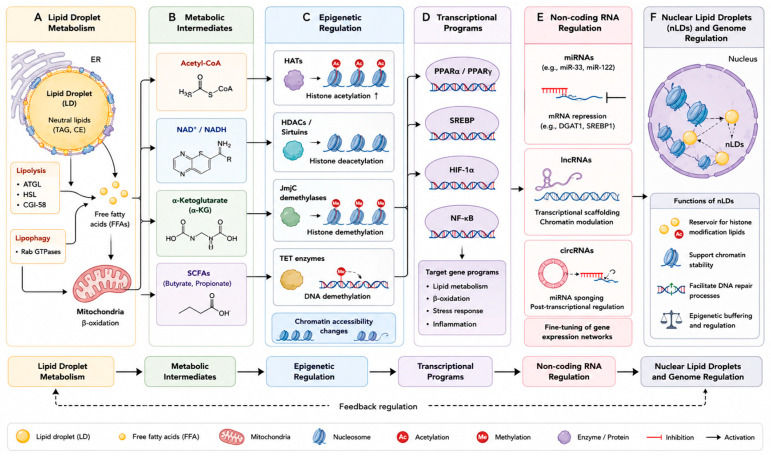
Metabolo-Epigenetic Coupling Mediated by Lipid Droplets. Lipid droplets (LDs) integrate metabolic flux with epigenetic remodeling through the regulated mobilization of lipid-derived metabolites. Lipolysis and lipophagy release fatty acids that undergo mitochondrial and peroxisomal β-oxidation to generate key metabolic intermediates, including acetyl-CoA, NAD+, α-ketoglutarate (α-KG), and short-chain fatty acids (SCFAs). These metabolites function as essential substrates or cofactors for chromatin-modifying enzymes, including histone acetyltransferases (HATs), sirtuin-dependent histone deacetylases (HDACs), TET DNA demethylases, and JmjC-domain histone demethylases. Through this metabolic provisioning, LD dynamics directly regulate histone acetylation, DNA methylation, chromatin accessibility, and transcriptional rewiring. Downstream transcriptional programs govern inflammation, hypoxia adaptation, ferroptosis resistance, tumor progression, and metabolic plasticity. Collectively, LDs act as upstream metabolic gatekeepers linking cellular lipid homeostasis with epigenetic adaptation and disease progression. This figure was created in BioRender. Yang, C. (2026). https://BioRender.com/xi9jnke; accessed on 22 March 2026.

Cellular metabolism and epigenetic modifications are coupled, as chromatin-modifying enzymes rely on intermediate metabolites as donor substrates or essential cofactors [88,89]. Lipid droplets (LDs) serve as metabolic regulators that control the availability of these nuclear cofactor pools by regulating intracellular lipid storage and mobilization [90]. As illustrated in Figure 3, LD-derived metabolites couple cellular lipid metabolism to epigenetic remodeling and transcriptional adaptation.

During active lipolysis, LD-derived fatty acids undergo mitochondrial or peroxisomal β-oxidation to generate a substantial flux of acetyl-CoA (Figure 3) [91]. This transiently expands the nucleocytoplasmic acetyl-CoA pool, providing the essential acetyl donor substrate for histone acetyltransferases (HATs) to promote histone hyperacetylation and maintain transcriptionally permissive chromatin configurations [92]. Notably, this lipid-driven epigenetic remodeling selectively targets distinct gene loci depending on the cellular context [18]. By enriching specific epigenetic activation marks, such as histone H3 lysine 27 acetylation (H3K27ac), LD-derived acetyl-CoA promotes the transcription of disease-associated genes [93]. In malignant cells, for example, enhanced LD breakdown hyperacetylates the promoter and enhancer regions of core oncogenes, such as MYC and CCND1 (Cyclin D1), thereby sustaining a proliferative state. Similarly, within activated inflammatory macrophages, this lipid-driven acetyl-CoA flux uncoils the chromatin architecture surrounding pro-inflammatory loci, triggering the expression of cytokines such as IL-1β and IL-6 [72].

Conversely, when fatty acid oxidation is suppressed, or during rapid lipid synthesis, alterations in the cellular NAD+/NADH ratio modulate the enzymatic activity of sirtuins (specifically SIRT1 and SIRT6), which are NAD+-dependent histone deacetylases [94,95]. Furthermore, fatty acid mobilization from LDs influences tricarboxylic acid (TCA) cycle flux, regulating α-ketoglutarate (α-KG) levels [96]. As an essential cofactor for Jumonji C domain-containing (JmjC) histone demethylases and Ten-Eleven Translocation (TET) DNA demethylases, fluctuations in α-KG link LD homeostasis with the removal of repressive histone and DNA methylation marks (Figure 3) [97]. Through this metabolic regulation, LDs function as upstream regulators of transcriptional plasticity and cellular identity. Moreover, alterations in the embryonic or postnatal nutrient environment upregulate the expression of genes involved in LD formation (e.g., Cidec) in the liver by promoting local histone acetylation and recruiting the epigenetic reader BRD4 across the gene body [18].

To systematically synthesize the interplay between lipid compartmentalization and genomic regulation, it is critical to recognize LDs not merely as storage units, but as dynamic metabolic drivers of the epigenome [73]. The LLPS at the LD-endoplasmic reticulum interface ensures the rapid, regulated mobilization of specific lipid pools during cellular stress. This precisely controlled catabolism channels intermediate metabolites directly to the nucleus, establishing a robust LD-epigenetic axis. The availability of these substrates acts as a rate-limiting step for various nuclear enzymes. Table 2 explicitly delineates the direct biochemical interactions between LD-generated metabolites and their corresponding chromatin-modifying enzymes. This mapping concentrates purely on the metabolic-epigenetic axis, demonstrating how the availability of specific intermediate metabolites triggers distinct epigenetic alterations. Complex disease phenotypes and specific transcriptional targets have been deliberately omitted from this table to prevent redundancy with Figure 3.

### 5.2. Fatty Acids and Chromatin Regulation

Beyond serving as an energy reserve, specific fatty acid species and lipid-derived signaling molecules act as modulators of chromatin architecture. Short-chain fatty acids (SCFAs), such as butyrate and propionate produced by gut microbiota, act as endogenous pan-HDAC inhibitors (Figure 3) [98]. By binding to the catalytic pockets of Class I and Class II HDACs, these fatty acids promote global transcriptional activation and re-activate silenced tumor suppressor genes [99].

Furthermore, recent lipidomic and biophysical analyses demonstrate that the fatty acid saturation composition of the cell influences the structural integrity of the nuclear envelope [100]. Shifts in the ratio of saturated to monounsaturated fatty acids (MUFAs) alter the fluidity of the nuclear membrane and reorganize lamina-associated domains (LADs). This structural remodeling repositions heterochromatin toward or away from the nuclear periphery, thereby modulating transcription factor accessibility and inducing persistent alterations in gene expression independent of signal transduction pathways.

### 5.3. LDs and Transcriptional Signaling Networks

LD metabolism is integrated into a multilayered feedback loop with distinct families of lipid-sensitive transcription factors that coordinate metabolic adaptation, cellular survival, and inflammatory defense (Figure 3).

PPARs (Peroxisome Proliferator-Activated Receptors): These nuclear receptors function as lipid sensors. Lipolysis of LDs by adipose triacylglycerol lipase (ATGL) releases long-chain polyunsaturated fatty acids (LC-PUFAs) and eicosanoid precursors that act as endogenous ligands for PPAR-α and PPAR-γ [101,102,103]. This binding triggers the transcription of genes involved in peroxisomal and mitochondrial β-oxidation, creating a feedback loop that balances lipid storage with energy expenditure [7].

SREBPs (Sterol Regulatory Element-Binding Proteins): SREBPs are key transcriptional regulators of cholesterol and fatty acid synthesis. When neutral lipids accumulate to high levels within the endoplasmic reticulum (ER) membrane, the SREBP cleavage-activating protein (SCAP)-SREBP complex is retained within the ER [104]. This prevents its translocation to the Golgi apparatus for proteolytic activation, establishing negative feedback inhibition of de novo lipogenesis [72].

HIF-1α (Hypoxia-Inducible Factor 1α): Under hypoxic stress, HIF-1α represses mitochondrial oxidative phosphorylation and upregulates lipid uptake transporters and hypoxia-inducible lipid droplet-associated protein (HILPDA/HIG2) [105]. This coordinated shift channels free fatty acids into triacylglycerols within LDs, protecting the hypoxic cell from lipid peroxidation and lipotoxicity [106].

YAP/TAZ and NF-κB: LDs are integrated into mechanosensitive and inflammatory signaling networks. Changes in cellular mechanical tension disrupt LD-cytoskeletal anchoring, modulating the nuclear translocation of YAP/TAZ to regulate proliferation [107]. Concurrently, during infection or metabolic inflammation, LDs act as platforms for the assembly of eicosanoids, promoting NF-κB activation and regulating innate immune signaling [108].

### 5.4. Non-Coding RNAs and LD Regulation

The post-transcriptional landscape of lipid metabolism is controlled by a network of non-coding RNAs (ncRNAs), including microRNAs (miRNAs), long non-coding RNAs (lncRNAs), and circular RNAs (circRNAs) (Figure 3) [109]. Specific miRNAs, such as miR-33 and miR-122, target the 3′ untranslated regions (UTRs) of transcripts encoding LD biogenesis enzymes (e.g., DGAT1/2) and lipolytic factors (e.g., ATGL, hormone-sensitive lipase), regulating the rate of lipid deposition [110].

Conversely, lncRNAs and circRNAs frequently function as competitive endogenous RNAs (ceRNAs) that sequester these regulatory miRNAs from their target transcripts [111]. For instance, stress-induced upregulation of specific lncRNAs can relieve the miRNA-mediated repression of perilipin proteins (PLINs), leading to rapid LD expansion. This ncRNA-LD axis forms a sensitive regulatory circuit that is frequently dysregulated in metabolic pathologies, including MASLD and malignant metabolic reprogramming [112].

### 5.5. Nuclear Lipid Droplets

An emerging area in organelle biology is the characterization of nuclear lipid droplets (nLDs), which exist as structurally distinct entities within the nucleoplasm rather than the cytoplasm (Figure 3) [113]. Arising primarily via budding from the inner nuclear membrane (INM), nLDs possess a distinct lipidomic profile rich in phosphatidylcholine and are encapsulated by a nuclear surface proteome including PLIN3 and lipid enzymes [113].

Rather than serving merely as storage depots, nLDs establish a functional interface with chromatin. They act as intranuclear lipid reservoirs that buffer the concentration of lipotoxic fatty acids inside the nucleus, thereby preserving genomic stability and protecting replication forks under oxidative stress [114]. Furthermore, nLDs serve as sites of epigenetic regulation; by localizing lipid-modifying enzymes and structural proteins near chromatin boundaries, they provide a localized pool of acetyl and acyl groups for histone modifications [17].

Recent perspectives establish that excessive nuclear lipid deposition is a key hallmark of aging. When nLDs aggressively build up around the nuclear lamina, they profoundly disrupt chromatin organization, DNA repair, and gene homeostasis, thereby accelerating cellular senescence [20]. Ultimately, current observations confirm that lipid droplets act as cohesive metabolic-nuclear hubs. They actively manage genomic stability, epigenetic shifts, and transcriptional flexibility via precise, metabolite-directed biochemical pathways, which are structurally cataloged in Table 2. Representative LD-associated proteins exhibiting LLPS-related properties are summarized in Table 3.

## 6. Lipid Droplets in Cellular Stress Adaptation

Lipid droplets (LDs) are no longer considered merely static fat storage depots; rather, they have emerged as dynamic organelles that coordinate cellular adaptation to environmental stresses [115]. By remodeling their lipid composition, recruiting specific regulatory proteins, and forming extensive contact sites with other organelles (such as the mitochondria, endoplasmic reticulum, and lysosomes), LDs maintain metabolic homeostasis and regulate cell fate under pathological conditions [12].

### 6.1. Nutrient Stress

Under conditions of nutrient deprivation, LDs function as energy reservoirs that maintain cellular survival. The mobilization of stored triacylglycerols proceeds through two coordinated pathways: cytosolic lipolysis (mediated by lipases such as ATGL and HSL) and selective autophagy, termed lipophagy [116]. Recent studies indicate that spatial regulation of these processes is essential. During starvation, LDs form physical contact sites with mitochondria (mediated by tethering proteins such as PLIN5) [117]. This spatial proximity ensures that released free fatty acids (FFAs) are transferred to mitochondria for β-oxidation and ATP production [1]. This transfer enhances energy production and prevents the accumulation of free fatty acids in the cytosol, thereby preventing lipotoxicity during metabolic crises [118].

### 6.2. Hypoxia

Hypoxia triggers a metabolic shift driven by hypoxia-inducible factors (HIF-1α and HIF-2α). Under low oxygen tension, mitochondrial electron transport becomes inefficient, risking the generation of reactive oxygen species (ROS) [119]. As an adaptive response, HIF signaling downregulates fatty acid oxidation (e.g., via CPT1 repression) while upregulating lipid uptake (via CD36 and FABPs) and de novo LD biogenesis. By diverting lipids away from the mitochondria and sequestering them into LDs, cells store energy without exacerbating oxidative stress [120]. This HIF-dependent LD accumulation has been identified as a survival mechanism in solid tumors, such as clear cell renal cell carcinoma, allowing cancer cells to survive in hypoxic microenvironments [121].

### 6.3. ER Stress

The endoplasmic reticulum (ER) is the primary site of lipid synthesis and protein folding. When disrupted by toxic lipids or misfolded proteins, the ER initiates the unfolded protein response (UPR) through sensors like IRE1α, PERK, and ATF6, which promote LD biogenesis [122]. Recent literature indicates the functional coupling between the ER and LDs at membrane contact sites regulated by proteins such as seipin [123,124]. During ER stress, LDs act as reservoirs that sequester lipotoxic species (such as ceramides and saturated FFAs) into neutral triacylglycerols [7]. Furthermore, misfolded ER proteins can be cleared and partitioned onto the LD surface for subsequent proteasomal degradation, thereby preserving ER membrane integrity and reducing ER stress [125].

### 6.4. Oxidative Stress and Ferroptosis

The role of LDs in oxidative stress has gained significant attention, particularly concerning ferroptosis—a form of regulated cell death driven by iron-dependent lipid peroxidation [126]. LDs protect cells from oxidative damage by dynamically altering their lipid profiles [8]. Specifically, enzymes such as ACSL3 and SCD1 promote the incorporation of monounsaturated fatty acids (MUFAs) into LDs [23]. This displaces oxidizable polyunsaturated fatty acids (PUFAs) from the plasma membrane, sequestering them within the hydrophobic core of the LDs where they are protected from ROS. Consequently, increased LD abundance is recognized as a signature of ferroptosis resistance, conferring a survival advantage to malignant cancer cells and protecting tissues from inflammatory injury [127,128].

### 6.5. Viral Infection, Inflammation, and Immunity

LDs represent an intersection between host metabolism and pathogen replication. Many viruses, including SARS-CoV-2, Hepatitis C Virus (HCV), and Dengue virus, exploit host lipid metabolism to induce extensive LD accumulation [129,130]. These pathogens utilize the LD surface as a site for viral encapsidation and replication [131,132]. In host cells, LDs serve as platforms for innate immune responses [133,134]. In immune cells such as macrophages, LDs recruit antimicrobial proteins (such as viperin) and store arachidonic acid. Upon inflammatory stimulation, this arachidonic acid is mobilized by enzymes residing on the LD surface (e.g., COX-2) to synthesize eicosanoids and prostaglandins, which in turn amplify inflammatory signaling, cytokine production, and immune cell polarization [135].

Collectively, these insights establish LDs as regulators of stress adaptation, linking lipid metabolism with cell survival, death, and immunity [136].

## 7. Lipid Droplets in Human Disease

**Figure 4:**
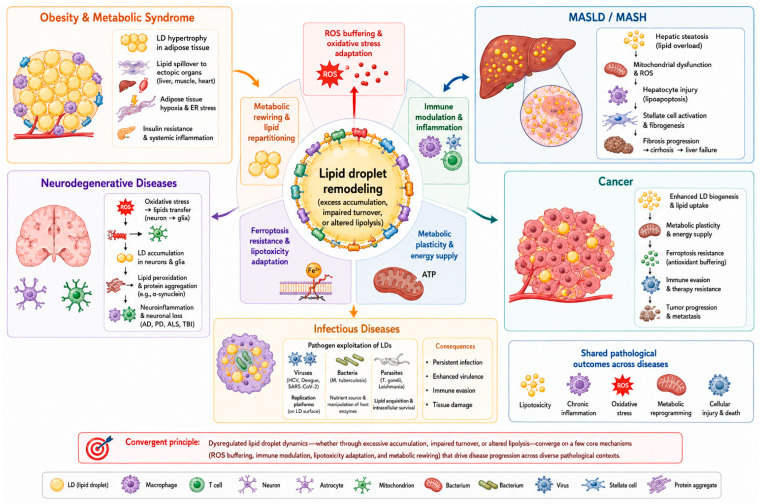
Pathological Remodeling of Lipid Droplets Across Human Diseases. Aberrant lipid droplet (LD) remodeling represents a conserved adaptive mechanism underlying diverse human diseases. In obesity and metabolic syndrome, excessive LD expansion and lipid spillover promote insulin resistance and chronic inflammation. In metabolic dysfunction-associated steatotic liver disease (MASLD/MASH), persistent lipid accumulation drives oxidative stress, mitochondrial dysfunction, and hepatocellular injury. In neurodegenerative disorders, LD accumulation in glial cells and interactions with aggregation-prone proteins contribute to neuroinflammation and proteotoxic stress. Cancer cells exploit LD remodeling to enhance metabolic plasticity, suppress ferroptosis, and evade anti-tumor immunity. During viral infection, pathogens hijack LD-associated pathways to support replication and inflammatory signaling. Despite disease-specific manifestations, these pathological states converge on common LD-associated mechanisms including ROS buffering, immune modulation, lipotoxicity adaptation, and metabolic rewiring. Solid colored arrows originating from the center indicate the diverse mechanistic pathways driven by lipid droplet remodeling; black downward arrows within specific panels represent the sequential cascade of pathological events.

As dynamic regulatory organelles, lipid droplets (LDs) integrate metabolic homeostasis and cellular stress responses. Dysregulation of LD biogenesis, maturation, or lipolysis disrupts systemic lipid partitioning, culminating in pathological conditions. Recent studies indicate that it is not the presence of LDs themselves, but rather the exhaustion of their buffering capacity that precipitates disease [3]. The pathological remodeling of LDs across human diseases is summarized in Figure 4.

### 7.1. Obesity and Metabolic Syndrome

Obesity is characterized by the pathological expansion of white adipose tissue, wherein adipocytes exhibit pronounced LD hypertrophy (Figure 4) [137]. When energy surplus exceeds the storage capacity of adipocyte LDs, a phenomenon known as “lipid spillover” occurs [1]. This results in ectopic lipid deposition in non-adipose tissues, such as skeletal muscle and the heart [21]. At the molecular level, impaired regulation of LD-associated proteins (e.g., perilipins) and defective lipolysis lead to the accumulation of lipotoxic intermediates, including diacylglycerols and ceramides [138]. These lipotoxic species interfere with insulin receptor signaling pathways, promoting peripheral insulin resistance [72]. Furthermore, hypertrophic LDs induce cellular stress and hypoxia within adipose tissue, triggering macrophage infiltration and the secretion of pro-inflammatory cytokines, contributing to chronic, low-grade inflammation (meta-inflammation) characteristic of metabolic syndrome [3].

### 7.2. MASLD and MASH (Formerly NAFLD and NASH)

Excessive hepatic LD accumulation, or steatosis, is the defining hallmark of metabolic dysfunction-associated steatotic liver disease (MASLD)—recently renamed from non-alcoholic fatty liver disease (NAFLD) to better reflect its systemic metabolic origins [139]. In the early stages, hepatic LDs act protectively by sequestering free fatty acids into inert triacylglycerols. However, persistent lipid overload overwhelms this buffering system (Figure 4) [140]. The unabated influx and subsequent oxidation of fatty acids exacerbate mitochondrial dysfunction and endoplasmic reticulum (ER) stress, driving the generation of reactive oxygen species (ROS) [141]. This lipotoxic microenvironment promotes hepatocellular apoptosis (lipoapoptosis) and provokes the activation of hepatic stellate cells, marking the critical pathophysiological transition from simple steatosis to metabolic dysfunction-associated steatohepatitis (MASH, formerly non-alcoholic steatohepatitis or NASH), which ultimately progresses to liver fibrosis and cirrhosis [142].

### 7.3. Neurodegenerative Diseases

The brain is enriched in lipids, and its metabolic homeostasis relies on cell-to-cell coupling [143]. Recent findings have identified LD accumulation as a pathogenic feature in Alzheimer’s disease (AD), Parkinson’s disease (PD), and amyotrophic lateral sclerosis (ALS) (Figure 4) [144,145]. Under oxidative stress, hyperactive neurons transfer toxic, peroxidized lipids to neighboring astrocytes and microglia, which package these lipids into LDs as a neuroprotective mechanism [146]. However, during aging or persistent stress, this glial LD buffering capacity declines, leading to lipotoxicity and neuroinflammation. Furthermore, genetic risk factors are linked to LD biology; for instance, the APOE4 allele in AD disrupts glial lipid transport and exacerbates LD accumulation [147]. In PD, phase-separated protein aggregates, such as α-synuclein, associate with the LD surface, disrupting lipid metabolism and accelerating neurodegeneration [148]. Beyond classical neurodegenerative diseases, in acute pathological states such as traumatic brain injury (TBI), hyperacetylation of fatty acid synthase (FASN) drives extensive LD accumulation in microglia [149]. This reprograms cellular metabolism and triggers a pro-inflammatory cytokine response, indicating an immunometabolic switch [149].

### 7.4. Cancer

Cancer cells typically undergo metabolic reprogramming wherein extensive LD remodeling is a hallmark of malignant tumor phenotypes (Figure 4). Elevated LD biogenesis serves multiple roles: it supports proliferation by providing a reservoir for ATP generation and membrane phospholipid synthesis, and it maintains redox homeostasis [150]. LDs confer metabolic plasticity, allowing cancer cells to survive nutrient deprivation and hypoxia within solid tumors [151].

By sequestering polyunsaturated fatty acids (PUFAs) into neutral triacylglycerols, LDs protect tumor cells from lipid peroxidation, mediating ferroptosis resistance [152]. Additionally, LD-derived lipid mediators (such as prostaglandin E2) are secreted into the tumor microenvironment to suppress T-cell function, facilitating immune evasion [153]. Consequently, dysregulated mobilization of these lipid stores correlates with tumor metastasis and therapy resistance, making LD metabolism a target for precision oncology [22,154].

### 7.5. Infectious Diseases

Intracellular pathogens frequently hijack host LDs to construct specialized niches for survival and replication (Figure 4). Numerous viruses—including Hepatitis C virus (HCV), Dengue virus, and SARS-CoV-2—directly target LDs, utilizing the phospholipid monolayer as a physical scaffold for viral genome replication and virion assembly [135]. Beyond viruses, intracellular bacteria such as *Mycobacterium tuberculosis* induce the formation of LD-rich “foam cells” in macrophages [155]. In this context, pathogens not only consume LDs as a rich carbon source but also manipulate LD-associated enzymes to alter the production of inflammatory eicosanoids [156]. This pathogen-driven LD remodeling subverts the host’s innate immune responses, promoting persistent infection and immune evasion [157]. To comprehensively synthesize the structural and functional variations of lipid droplets across major pathologies, Table 4 offers a descriptive disease-phenotype atlas. This summary focuses entirely on how abnormal LD behaviors—such as pathological expansion, accelerated formation, or unusual nuclear localization—present at the cellular level and determine specific functional results in various human diseases. Phenotypic traits have been intentionally isolated from the molecular signaling pathways in this table to eliminate overlap with Figure 4.

## 8. Therapeutic Opportunities Targeting LD Biology

Therapeutic interventions targeting lipid droplet (LD) biology have emerged from preclinical studies as clinically viable strategies. LDs integrate energy homeostasis, stress adaptation, and lipid signaling; therefore, selectively modulating their biogenesis, breakdown, or physical properties offers an approach to treating metabolic, oncological, and neurodegenerative diseases [158].

### 8.1. DGAT Inhibitors

Diacylglycerol O-acyltransferase 1 and 2 (DGAT1/2) catalyze the final and committed step of triacylglycerol (TG) synthesis, making them key targets for restricting LD biogenesis. In the context of metabolic dysfunction-associated steatotic liver disease (MASLD) and MASH, pharmacological inhibition of DGAT (e.g., via dual DGAT1/2 inhibitors or DGAT2-specific agents like ervogastat) reduces hepatic steatosis and mitigates subsequent fibrotic responses [159]. In oncology, disrupting TG synthesis via DGAT inhibition forces the cytosolic accumulation of unesterified free fatty acids, triggering lipotoxicity. By preventing cancer cells from sequestering lipids, DGAT inhibitors compromise tumor metabolic plasticity and deprive dividing cells of energy reserves required for survival under stress [160]. The clinical translation of these targets has yielded candidate drugs, particularly for the management of MASH. For instance, Ervogastat (PF-06865571), a DGAT2 inhibitor, has advanced into Phase 2 clinical trials (e.g., NCT05342045), being evaluated in combination with acetyl-CoA carboxylase (ACC) inhibitors to mitigate hepatic steatosis [161]. Similarly, antisense oligonucleotide (ASO) therapies targeting DGAT2, such as Fesomersen (IONIS-DGAT2Rx), have completed Phase 2 evaluations (NCT04608352), which supports the therapeutic suppression of TG synthesis in human metabolic disease [162].

### 8.2. Ferroptosis-Based Therapeutic Strategies

The recognition that LDs buffer cancer cells against lipid peroxidation has paved the way for novel ferroptosis-inducing therapies [163]. Because LDs safely sequester easily oxidizable polyunsaturated fatty acids (PUFAs) into neutral triacylglycerols, tumors exploit this mechanism to evade ferroptosis [164]. Therapeutic strategies are now focusing on disabling this lipid sink. For instance, inhibiting DGAT or stearoyl-CoA desaturase 1 (SCD1) restricts the storage of monounsaturated and polyunsaturated lipids in LDs, forcing PUFAs to mislocalize into the plasma membrane [93]. When combined with glutathione peroxidase 4 (GPX4) inhibitors (such as RSL3), this LD depletion strategy synergistically amplifies lipid peroxidation, demonstrating the potential in preclinical models to re-sensitize therapy-resistant solid tumors to ferroptosis-mediated cell death [165]. Moving beyond the preclinical stage, pharmacological disruption of lipid metabolism is now advancing into early clinical testing in oncology as a combinatorial strategy to bypass drug resistance [166]. For example, Denifanstat (TVB-2640), an inhibitor of fatty acid synthase (FASN), is currently undergoing Phase 2 clinical trials (NCT03179904) in combination with paclitaxel and trastuzumab for HER2-positive metastatic breast cancer, exploiting the metabolic dependencies of tumor lipid networks [167]. Furthermore, SCD1 inhibitors such as MTI-301 have entered early-phase clinical testing (NCT06911008) for advanced solid tumors, accelerating the translation of lipid-peroxidation and ferroptosis-sensitizing concepts into clinical oncology [168].

### 8.3. Targeting Phase-Separation Pathways

A significant advance in LD biology is the role of LLPS in LD biogenesis and protein targeting [13]. Many LD-associated proteins, including seipin and perilipins, contain intrinsically disordered regions (IDRs) that promote their condensation at the ER-LD interface [169]. In neurodegenerative diseases, pathogenic proteins such as α-synuclein undergo abnormal LLPS and co-assemble on the surface of LDs, accelerating pathological aggregation and organelle dysfunction [16]. Consequently, targeting the biophysical properties of these biomolecular condensates represents an emerging therapeutic frontier [7]. Small molecules designed to modulate the LLPS dynamics of LD-associated proteins may dissolve pathological aggregates or correct defective lipid partitioning in neurological and metabolic disorders [170].

### 8.4. Epigenetic Therapies Linked to LD Metabolism

The intersection of lipid metabolism and epigenetics—often termed “metabolo-epigenetics”—reveals that LDs directly govern nuclear transcriptional programs [171]. The lipolysis of LDs, primarily mediated by adipose triacylglycerol lipase (ATGL), generates a steady flux of fatty acids that undergo mitochondrial β-oxidation to produce acetyl-CoA [172]. This lipolysis-derived acetyl-CoA serves as a crucial substrate for histone acetyltransferases (HATs) [173]. In cancer and inflammatory macrophages, heightened LD breakdown sustains high levels of histone acetylation (e.g., H3K27ac), which drives the transcription of oncogenes and pro-inflammatory cytokines [174]. Pharmacologically inhibiting ATGL or CPT1 can starve the epigenetic machinery of acetyl-CoA, thereby suppressing oncogenic transcription [175]. Combining LD-targeting drugs with epigenetic modulators (such as HDAC inhibitors) is emerging as a potent strategy to overcome epigenetic drug resistance [19].

### 8.5. Nanomedicine and LD-Targeted Drug Delivery

The highly hydrophobic, neutral lipid core of LDs provides a unique sub-cellular target for nanomedicine. Recent advancements in nanotechnology have developed lipophilic nanocarriers and engineered prodrugs that selectively accumulate within LDs [176]. Notably, aggregation-induced emission luminogens (AIEgens) have been designed not only to precisely image LD dynamics in live cells but also to act as therapeutic agents. Upon targeted accumulation within tumor LDs, these nanoscale photosensitizers can be activated by specific wavelengths of light to generate reactive oxygen species (ROS) directly inside the lipid core [177]. In experimental settings, this targeted photodynamic therapy (PDT) oxidizes stored lipids from within, leading to severe structural damage to the LDs and promoting cell death in malignant tissue models. However, these approaches remain strictly experimental, and extensive preclinical validation regarding their systemic safety and delivery efficiency is required before clinical translation can be considered [178].

### 8.6. Challenges in Clinical Translation: Toxicity and Off-Target Effects

While targeting LD biology presents immense therapeutic potential, systemic intervention in lipid metabolism carries significant risks of off-target effects [179]. For example, forcefully inhibiting tumor LD biogenesis (e.g., via systemic DGAT inhibitors) could precipitate ectopic lipid deposition and severe lipotoxicity in healthy, non-target organs such as the heart or skeletal muscle [180]. Future therapeutic designs must prioritize targeted drug delivery systems, such as functionalized nanomedicines, to minimize these systemic adverse effects [181,182].

### 8.7. Landscape of Current and Emerging Therapeutic Interventions

Successfully applying our knowledge of lipid droplets to medical treatments demands a strategic focus on both the initial production of lipids and their subsequent metabolic cascades. We present a comprehensive overview of these targeted interventions in Table 5. This updated summary explicitly spans the entire developmental spectrum—from preliminary experimental models and preclinical studies to established clinical trials addressing cancer and metabolic dysfunctions.

## 9. Conclusions and Perspectives

### 9.1. A Paradigm Shift in Organelle Biology

Over the past decade, the understanding of lipid droplets (LDs) has evolved significantly. LDs are no longer viewed as inert cytosolic fat depots [4]. Instead, they have emerged as dynamic organelles that function in metabolic–epigenetic signaling [7]. By coordinating interactions among lipid metabolism, liquid–liquid phase separation (LLPS), stress adaptation, and transcriptional regulation, LDs regulate cell fate under microenvironmental fluctuations [3].

### 9.2. Pathological Implications of LD Rewiring

This interplay among LD remodeling, LLPS-driven protein recruitment, and epigenetic reprogramming provides cells with adaptive flexibility. This adaptive capacity is important in the pathogenesis of systemic and cellular disorders. In obesity and metabolic dysfunction-associated steatotic liver disease (MASLD), impaired LD buffering capacity triggers lipotoxicity and meta-inflammation [7]. Concurrently, in neurodegeneration, infection, and cancer, aberrant LD-mediated metabolic rewiring is exploited by stressed cells or pathogens to support proliferation, avoid host immune surveillance, and resist ferroptotic death [183]. Consequently, targeting LD vulnerabilities has emerged from a basic science concept to a translational strategy [184].

### 9.3. Unresolved Questions and Knowledge Gaps

Despite these advances, several important gaps remain to be addressed:

Biophysical Classification: The boundary between classical membrane-bound organelles and membraneless biomolecular condensates is becoming less distinct. It remains a subject of ongoing biophysical debate whether the initial budding of LDs and the assembly of their monolayer proteome should be classified and modeled under the physical rules of LLPS [7].

In Vivo LLPS Validation: Despite interest in LD-associated phase separation, current research faces methodological limitations. Many conclusions regarding the LLPS of LDs and proteins such as seipin are derived from in vitro reconstituted systems or overexpression models [185]. Validating these phase-separation dynamics under native physiological conditions in vivo remains technically challenging and requires further validation in future studies [186].

Nuclear Lipid Droplets (nLDs): The recent identification of nLDs has opened a new area of lipid biology. It is still unclear how nLDs interact with chromatin remodeling complexes, and how they supply lipid-derived metabolites (such as acetyl-CoA or signaling oxylipins) to regulate epigenetic patterns and gene transcription within the nucleus [20].

Spatiotemporal Heterogeneity: LDs are not a homogenous population. Understanding how LD heterogeneity—varying in size, lipidomic composition, and protein coat—contributes to disease progression at the single-organelle and single-cell level remains an analytical challenge [187].

### 9.4. Future Technological Frontiers

Addressing these questions will require the integration of next-generation analytical platforms. The advent of spatial lipidomics and single-cell metabolomics enables mapping of the lipidome of specific LD subpopulations within their native tissue architecture [188]. Furthermore, structural approaches such as in situ cryo-electron tomography (cryo-ET) enable visualization of the sub-nanometer molecular architecture of LD-organelle contact sites [189]. When combined with super-resolution live-cell imaging and synthetic fluorophores, these technologies enable real-time tracking of LD phase transitions and inter-organelle lipid fluxes [190].

Ultimately, decoding the mechanisms by which LDs coordinate metabolic flux, biophysical phase separation, and epigenetic regulation will expand our understanding of cellular adaptation and lead to targeted therapies for metabolic, infectious, and malignant diseases.

## Figures and Tables

**Table 1:** Core Lipid Droplet-Associated Proteins and Their Functions in LD Biogenesis, Remodeling, and Signaling.

Protein/Complex	Primary Localization	Core Function	Mechanistic Role in LD Biology	Disease Relevance
Seipin (BSCL2)	ER–LD junction	LD nucleation scaffold	Forms oligomeric cage to trap neutral lipids and direct LD budding	Congenital lipodystrophy, metabolic syndrome
FIT2	Endoplasmic reticulum	Lipid remodeling	Regulates DAG/acyl-CoA balance and ER membrane curvature	ER stress, steatosis
DGAT1/2	Endoplasmic reticulum	TG synthesis	Catalyze neutral lipid formation driving LD nucleation	Obesity, MASLD, cancer
ACAT1/2	Endoplasmic reticulum	Cholesteryl ester synthesis	Generate CE for LD core formation	Atherosclerosis
PLIN1	LD monolayer	Lipolytic barrier	Restricts ATGL access under basal conditions	Obesity, insulin resistance
PLIN5	LD–mitochondria interface	FA channeling	Couples LDs to mitochondria for β-oxidation	Cardiomyopathy, metabolic disease
ATGL	LD surface	Lipolysis initiation	Hydrolyzes TGs to release fatty acids	Cachexia, lipotoxicity
CGI-58	LD surface	ATGL co-activation	Activates lipolysis following PLIN phosphorylation	Neutral lipid storage disease
HSL	LD surface	Neutral lipid hydrolysis	Mobilizes DAGs and TGs	Obesity, diabetes
CIDEA/CIDEC	LD–LD contact site	LD fusion	Drive LLPS-dependent lipid transfer and droplet coalescence	Adipocyte hypertrophy
ACSL4	ER and LD-associated membranes	PUFA activation	Sensitizes cells to ferroptosis	Cancer, ferroptosis
HILPDA	LD surface	Hypoxia adaptation	Promotes LD accumulation under hypoxia	Tumor progression
Rab18	LD contact sites	Organelle tethering	Regulates LD interaction with ER and autophagosomes	Warburg Micro syndrome
CCT	LD surface	Phosphatidylcholine synthesis	Stabilizes expanding LD monolayer	Lipotoxic stress
SCD1	ER membrane	MUFA synthesis	Modulates membrane fluidity and ferroptosis resistance	Cancer, obesity

**Table 2:** Lipid droplet-derived metabolites regulate epigenetic remodeling.

Metabolite	Source (LD-Linked Metabolism)	Target Enzymes	Epigenetic Modification	Functional Consequence
Acetyl-CoA	Fatty acid β-oxidation	Histone acetyltransferases (HATs)	Histone acetylation (e.g., H3K27ac)	Chromatin opening, transcriptional activation
NAD^+^/NADH	Mitochondrial oxidative metabolism	Sirtuins (Class III HDACs)	Histone deacetylation	Stress adaptation, metabolic rewiring
α-Ketoglutarate (α-KG)	TCA cycle modulation	JmjC & TET demethylases	Histone/DNA methylation	Epigenetic erasure, lineage regulation
Butyrate/SCFAs	Specialized lipid mobilization	Class I/II HDACs	HDAC inhibition	Global transcriptional activation

**Table 3:** LLPS-Related Properties of Lipid Droplet-Associated Proteins.

Protein	IDR Presence	LLPS Behavior	Functional Consequence	Experimental Evidence
CIDEA	Yes	Forms phase-separated condensates at LD interfaces	Drives LD fusion	In vitro and cellular imaging
CIDEC/FSP27	Yes	Stabilizes lipid transfer pores	Promotes unilocular LD formation	Cryo-EM and live-cell imaging
Seipin	Partial	Organizes nanoscale lipid domains	Controls LD nucleation	Structural biology studies
PLIN5	Predicted	Dynamic surface clustering	Regulates LD–mitochondria coupling	Fluorescence microscopy
HILPDA	Predicted	Hypoxia-responsive condensates	Promotes LD accumulation	Hypoxia models
ATGL complex	Partial	Dynamic enzymatic microdomains	Spatial lipolysis regulation	Biochemical studies
α-synuclein	Yes	Aggregates on LD surfaces	Disrupts lipid homeostasis	Neurodegeneration models
APOE4-associated complexes	Predicted	Alters lipid-protein phase behavior	Enhances glial LD accumulation	AD models

**Table 4:** Lipid droplet remodeling in human disease contexts.

Disease	Lipid Droplet Phenotype	Associated Cellular Features	Functional Outcome	Disease
Obesity & Metabolic Syndrome	Adipocyte LD hypertrophy	Excess lipid storage, insulin signaling disruption	Insulin resistance, metabolic dysfunction	Obesity & Metabolic Syndrome
MASLD/MASH	Hepatic steatosis with LD accumulation	ER stress, mitochondrial overload	Fibrosis progression, liver dysfunction	MASLD/MASH
Cancer	Increased LD biogenesis and remodeling	Enhanced lipid buffering capacity	Therapy resistance, survival advantage	Cancer
Neurodegeneration	Glial LD accumulation	Oxidative stress, protein aggregation	Neuronal dysfunction and loss	Neurodegeneration
Infectious Diseases	Pathogen-induced LD hijacking	Altered lipid trafficking, immune modulation	Persistent infection, immune evasion	Infectious Diseases
Aging	Nuclear LD accumulation (nLDs)	Chromatin instability, impaired repair	Cellular senescence, aging phenotype	Aging

**Table 5:** Therapeutic Strategies Targeting Lipid Droplet Metabolism and Phase-Separation Pathways.

Therapeutic Target	Representative Strategies	Primary Mechanism	Biomarker/Readout	Clinical Barrier	Development Status
DGAT1/2	DGAT inhibitors	Suppress TG synthesis and LD biogenesis	Hepatic lipid content	Gastrointestinal toxicity	Early clinical (e.g., Phase 2 for MASH)/Preclinical (Oncology)
ATGL	ATGL inhibitors	Reduce lipolysis and FA release	Circulating FFAs	Systemic metabolic effects	Preclinical
FASN	TVB-2640	Block de novo lipogenesis	Tumor lipid metabolism	Metabolic compensation	Early clinical
SCD1	SCD1 inhibitors	Reduce MUFA synthesis	Ferroptosis sensitivity	Lipotoxicity	Early clinical/Preclinical
ACSL4	ACSL4 targeting	Modulate PUFA incorporation	Lipid peroxide levels	Ferroptosis-associated injury	Experimental
Lipophagy	Autophagy activators	Enhance LD degradation	Autophagic flux	Non-specific autophagy effects	Experimental/Preclinical
HIF-1α/HILPDA	Hypoxia pathway inhibition	Prevent hypoxia-induced LD accumulation	Hypoxic markers	Tumor heterogeneity	Preclinical
GPX4/Ferroptosis	Ferroptosis inducers	Promote oxidative lipid damage	ROS/lipid peroxidation	Off-target toxicity	Early clinical/Preclinical
LLPS machinery	Condensate-disrupting molecules	Interfere with phase-separated LD domains	Protein condensate dynamics	Specificity challenges	Experimental
Immune-LD axis	COX-2 inhibitors	Suppress inflammatory lipid signaling	Eicosanoid production	Immune suppression	Clinically established (for general inflammation)/Experimental (for specific LD targeting)

## Data Availability

No new data were created or analyzed in this study. Data sharing is not applicable to this article.

## Abbreviations

The following abbreviations are used in this manuscript:

LDs	Lipid droplets
TG/TGs	triacylglycerols
CEs	Cholesteryl esters
CIDE	cell death-inducing DFFA-like effector
ER	endoplasmic reticulum
ROS	reactive oxygen species
LLPS	liquid–liquid phase separation
IDRs	intrinsically disordered regions
SAM	S-adenosylmethionine
MASLD	metabolic dysfunction-associated steatotic liver disease
MASH	metabolic dysfunction-associated steatohepatitis
DGAT	diacylglycerol acyltransferases
ACAT	acyl-CoA:cholesterol acyltransferases
FIT	fat-inducing transcript
PC	phosphatidylcholine
PE	phosphatidylethanolamine
CCT	CTP: phosphocholine cytidylyltransferase
DAG	diacylglycerols
PLIN	perilipin
ATGL	adipose triacylglycerol lipase
HSL	hormone-sensitive lipase
COPI	coat protein complex I
PUFAs	polyunsaturated fatty acids
MCSs	membrane contact sites
UPR	unfolded protein response
ATGs	autophagy-related proteins
NAFLD	non-alcoholic fatty liver disease
ACSL4	acyl-CoA synthetase long-chain family member
LAMs	lipid-associated macrophages
α-KG	α-ketoglutarate
HDACs	histone deacetylases
JmjC	Jumonji C domain-containing
SCFAs	short-chain fatty acids
PPARs	peroxisome proliferator-activated receptors
SREBPs	sterol regulatory element-binding proteins
ncRNAs	non-coding RNAs
miRNAs	microRNAs
lncRNAs	long non-coding RNAs
UTRs	untranslated regions
TBI	traumatic brain injury
HCV	Hepatitis C virus
ACC	acetyl-CoA carboxylase
ASO	antisense oligonucleotide
SCD1	stearoyl-CoA desaturase 1
GPX4	glutathione peroxidase 4
FASN	fatty acid synthase
HATs	histone acetyltransferases
AIEgens	aggregation-induced emission luminogens
nLDs	nuclear lipid droplets
